# Cardiovascular metabolic risk factors and glomerular filtration rate: a rural Chinese population study

**DOI:** 10.1186/s12944-016-0346-9

**Published:** 2016-10-12

**Authors:** Wei Zheng, Geng Qian, Wenjun Hao, Xiaodong Geng, Quan Hong, Guangyan Cai, Xiangmei Chen, Di Wu

**Affiliations:** 1 Department of Nephrology, Chinese PLA General Hospital, Chinese PLA Institute of Nephrology, State Key Laboratory of Kidney Diseases, National Clinical Research Center for Kidney Diseases, Beijing, China; 2 Department of Cardiology, Chinese PLA General Hospital, Beijing, China; 3 Chinese PLA General Political Department Huang Si First Clinic for Retired Cadres, Beijing, China

**Keywords:** MetS, eGFR, Rural Chinese population, Cross-sectional survey

## Abstract

**Methods:**

A total of 2426 study subjects from rural China aged 35 years and above (934 men and 1492 women) were enrolled in a cross-sectional survey. The eGFR calculation was based on the Modification of Diet in Renal Disease (MDRD) equation. The strength of the association between cardiovascular metabolic risk factors and eGFR was analyzed using a linear regression model.

**Results:**

Cardiovascular metabolic risk factors, including age, body weight, waist circumference, fasting plasma glucose (FPG), creatinine (Cr), high-density lipoprotein-cholesterol (HDL-C), total cholesterol (TC), triglyceride (TG), systolic pressure, and diastolic pressure, were associated with eGFR. Additionally, the eGFR level gradually decreased and showed a linear trend with the increase in metabolic syndrome risk factors.

**Conclusion:**

Metabolic risk factors are correlated with a reduction in renal function and CKD.

## Background

Chronic kidney disease (CKD) and cardiovascular diseases severely threaten human health and life. Compelling evidence shows that CKD and cardiovascular diseases usually occur together. These two diseases have a common pathophysiological basis. Traditional cardiovascular risk factors, including hypertension, hyperuricemia, advanced age, diabetes mellitus, increased low-density lipoprotein-cholesterol (LDL-C), smoking, and alcoholism, are also risk factors for kidney diseases [1–5]. However, the association between cardiovascular metabolic risk factors and CKD is uncertain for Chinese individuals.

In addition, the aging of the population and economic development have facilitated changes in the diet and lifestyle of the Chinese population. Therefore, the prevalence of metabolic syndrome (MetS), which is primarily characterized by obesity, hypertension, abnormal glucose metabolism, blood lipid disorders, hyperinsulinemia, and hyperuricemia, has gradually increased. Previous studies showed that MetS was a common risk factor for cardiovascular diseases and CKD [6] and that some MetS factors were associated with the development of CKD.

However, no previous study has investigated the association between cardiovascular metabolic risk factors and kidney function levels in a rural Chinese population. Therefore, in the present study we examined the association between metabolic risk factors and kidney function in a rural Chinese population. We tested whether the cardiovascular metabolic risk factors were associated with the estimated glomerular filtration rate (eGFR) and whether MetS is also associated with an eGFR decrease.

## Methods

### Study population

The study subjects were from the China Stroke Primary Prevention Trial (CSPPT, clinical trial number NCT00794885). The data were collected from residents of rural areas in Lianyungang City of Jiangsu Province and Anqing City of Anhui Province, China. The inclusion criterion was an age of 35–90 years. The exclusion criteria included a reported history of myocardial infarction, stroke, heart failure, cancer, or serious mental disorders; unwillingness to participate; and difficulty completing the survey. The study conformed to the Declaration of Helsinki and was approved by the ethics committee of the Institute of Biomedicine, Anhui Medical University, Hefei, China.

A stratified random cross-sectional survey was performed using the cluster sampling method. In total, 2426 people aged from 39 to 88 (63 ± 7) years were screened. All study subjects signed informed consent forms. The results were used for public data analysis and the purposes of this study.

### Data collection

All study subjects received physical examinations. A questionnaire was used to collect lifestyle information and disease history. The smoking conditions were classified as current, previous, and non-smoking. The body weight and height were measured in the morning with the participants wearing light clothing and no shoes. The BMI was calculated using the equation BMI = body weight (kg) divided by the square of the body weight (kg/m^2^). eGFR was calculated using the Modified Diet in Renal Disease (MDRD) equation. The blood pressure of the study subjects was measured using an Omron automatic digital blood pressure monitor (Omron HEM705IT device; OmronHealth Care) according to the American Heart Association protocol. The study subjects rested for at least 5 min and assumed a supine position. The right arm was placed at the same level as the heart. The measurement was performed 3 times at 2-min intervals. The mean value was calculated.

Each study subject fasted for 12–16 h prior to the collection of a venous blood sample. Serum or plasma samples were separated 30 min after collection, stored in a −70 °C freezer, and sent together for measurements.

### Blood test

Serum creatinine (Cr), urea nitrogen (UN), uric acid (Ua), fasting plasma glucose (FPG), total cholesterol (TC), triglyceride (TG), high-density lipoprotein-cholesterol (HDL-C), and alanine aminotransferase (ALT) levels were determined using an automatic analyzer (Roche). The homocysteine level was determined using high-performance liquid chromatography (HPLC). eGFR was calculated using the MDRD equation.

### Statistical analyses

We examined the associations of the cardiometabolic risk factors with eGFR using a general linear model. Standardized regression coefficients for eGFR on metabolic risk factors were obtained from a standardized linear regression model where we treated eGFR as the dependent variable and cardiometabolic risk factors as independent variables. We further tested the associations between MetS and eGFR using general linear models after adjustment for potential confounders (age, gender). All reported *P* values are nominal and two sided. Statistical analyses were performed in SAS 9.3 (SAS Institute, Cary, NC, USA).

## Results

### Basic characteristics of the study population

The study included 2426 subjects, including 934 men and 1492 women. The age, waist circumference, BMI, FPG, HDL-C, TC, TG, Cr, serum albumin, UN, and blood pressure (including systolic pressure and diastolic pressure) were assessed for data analysis. The general information is provided in Table 1.

**Table 1:** Characteristics of the study sample (mean ± S.D.)

Characteristics	Men	Women
Number	934	1492
Age (years)	63.95 ± 7.20	61.63 ± 7.47
Waist (cm)	77.45 ± 10.13	79.38 ± 10.34
Body mass index (kg/m)	21.94 ± 3.24	23.35 ± 3.63
Fasting plasma glucose (mg/dl)	6.18 ± 1.99	6.14 ± 1.96
HDL cholesterol (mg/dl)	1.51 ± 0.39	1.47 ± 0.34
Total cholesterol (mg/dl)	4.92 ± 0.92	5.29 ± 1.06
Triglycerides (mg/dl)	1.37 ± 0.89	1.81 ± 1.25
Systolic blood pressure (mmHg)	125.05 ± 19.15	126.05 ± 18.97
Diastolic blood pressure (mmHg)	79.70 ± 10.69	78.77 ± 9.72
Creatinine (μmol/L)	87.66 ± 18.05	73.00 ± 15.36
Glomerular filtration rate	103.82 ± 21.12	96.19 ± 20.44
Albumin (g/L)	47.84 ± 3.55	48.26 ± 3.53
Blood urea nitrogen (mmol/L)	6.36 ± 1.84	5.84 ± 1.68

### Association of cardiovascular metabolic risk factors with eGFR

CKD (chronic kidney disease) is defined as eGFR < 60 ml/min per 1.73 m^2^ (MDRD). The 2426 study subjects were grouped accordingly as eGFR ≥ 90 ml/min/1.73 m^2^ (1611 cases), eGFR = 60–89 ml/min/1.73 m^2^ (773 cases), and eGFR < 60 ml/min/1.73 m^2^ (42 cases) prior to the correlation analysis. The results showed that the risk factors age, waist circumference, Cr, HDL-C, TC, TG, systolic pressure, and diastolic pressure of the population with lower values of eGFR were significantly higher than those of the population with higher values of eGFR (*P* for trend: <0.05). In contrast, eGFR and body weight showed a negative correlation (P for trend: 0.0026) (Table 2).

**Table 2:** Metabolic risk factors according to eGFR

Characteristic	eGFR ≥ 90 (*n* = 1611)	eGFR 89–60 (*n* = 773)	eGFR < 60 (*n* = 42)	P for trend
Age (years)	61.54 ± 7.21	64.27 ± 7.52	67.88 ± 7.22	<.0001
Weight (kg)	56.56 ± 9.97	55.41 ± 9.92	54.74 ± 11.44	0.0026
Waist (cm)	78.41 ± 10.20	79.08 ± 10.47	79.19 ± 10.87	0.0191
Fasting plasma glucose (mg/dl)	6.08 ± 1.93	6.22 ± 1.86	8.00 ± 3.85	0.0609
Creatinine (μmol/L)	72.05 ± 11.80	88.86 ± 12.31	143.58 ± 51.86	<.0001
Glomerular filtration rate	109.66 ± 16.82	79.85 ± 7.51	49.61 ± 10.64	<.0001
HDL cholesterol (mg/dl)	1.47 ± 0.34	1.51 ± 0.37	1.59 ± 0.69	0.0021
Total cholesterol (mg/dl)	5.03 ± 0.94	5.33 ± 1.10	6.03 ± 1.63	<.0001
Triglycerides (mg/dl)	1.58 ± 1.12	1.73 ± 1.18	2.21 ± 1.02	0.0021
Systolic blood pressure (mmHg)	124.48 ± 18.42	127.73 ± 19.88	133.05 ± 22.06	0.0144
Diastolic blood pressure (mmHg)	78.71 ± 9.97	79.92 ± 10.34	80.65 ± 10.83	<.0001

**Fig. 1:**
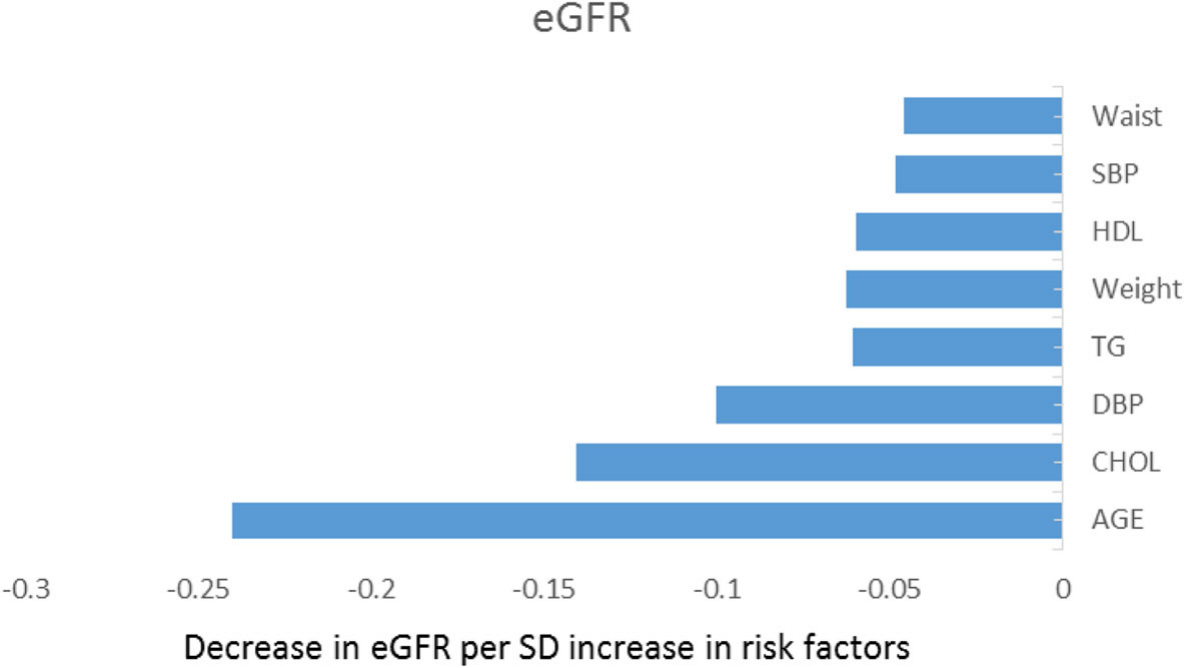
Standardized regression coefficients for eGFR vs metabolic risk factors. The decrease in standard deviations of eGFR per standard deviation increase in the metabolic risk factors ranged from 0.05 (waist) to 0.25 (age)

Standardized linear regression analysis was performed on the 2426 study subjects. eGFR was used as a dependent variable, and age, body weight, waist circumference, HDL-C, TC, TG, systolic pressure, and diastolic pressure were individually introduced into the model. The results showed that the eGFR gradually decreased as the metabolic risk factors increased. The decrease in the standard deviations of eGFR per standard deviation of the increase in the metabolic risk factors ranged from 0.05 (waist) to 0.25 (age) (Fig. 1).

### Association between MetS and eGFR

**Fig. 2:**
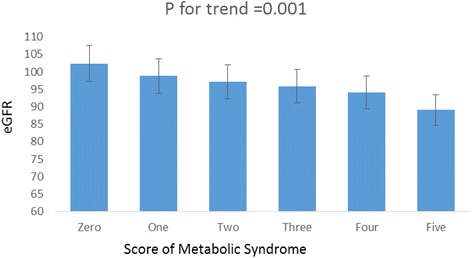
Metabolic syndrome and eGFR

MetS was defined as the presence of at least 3 of 5 risk factors (waist circumference, TG, HDL-C, blood pressure, and fasting plasma glucose). Specifically, the risk factors were enlarged waist circumference according to population-specific and country-specific criteria; TG ≥ 150 mg/dl; HDL-c < 40 mg/dl in men and <50 mg/dl in women; systolic blood pressure ≥ 130 mm Hg or diastolic blood pressure ≥ 85 mm Hg; and FPG > 100 mg/dl. Patients taking medication to manage hypertriglyceridemia, low HDL-c, hypertension or hyperglycemia were also included [7]. The results showed that the eGFR gradually and linearly decreased as the number of MetS risk factors increased (Fig. 2).

## Discussion

We mainly found that cardiovascular metabolic risk factors correlated with the eGFR. The eGFR gradually decreased as the metabolic syndrome risk factors increased in the rural Chinese population.

A systemic analysis of the European population showed that increased blood pressure and TG and decreased LDL-C, obesity, and glucose metabolic disorders were significantly associated with CKD development (eGFR < 60 ml/min per 1.73 m^2^) and eGFR reduction. Additionally, the strength of this correlation increased as the number of the above factors increased [8]. Our study results in the Chinese population were consistent with previous studies showing a negative correlation between eGFR and cardiovascular metabolic risk factors, such as hypertension, hypercholesterolemia, and hypertriglyceridemia, as well as reduced HDL-C hyperlipidemia and obesity. Studies on a European population showed that hypertriglyceridemia and a low HDL-C level were both risk factors for eGFR reduction in the non-kidney disease population and for CKD progression in kidney disease patients [9–11]. In the healthy population, age is the most important factor that causes a reduction in kidney function; an increase in blood pressure and in characteristics of metabolic disorders, such as high blood glucose and blood lipid lipids, are also correlated with age [12–14]. Our study found that age was associated with a reduction in eGFR. In addition, hypertension is one risk factor for mortality worldwide [15]. The studies by Kurella et al. and Rashidi et al. both showed that increased blood pressure plays an important role in the increase in the CKD incidence rate [16, 17], which was consistent with our study results. Our results showed that an increase in diastolic pressure had greater effects on the reduction in eGFR than an increase in systolic pressure.

Kurella et al. performed a 9-year prospective cohort study on adults without diabetes mellitus. In 2005, MetS was reported to be an independent risk factor for CKD development; the relative risk for CKD was higher among people with more abnormal MetS-associated factors. This study suggested that MetS is the major cause of CKD in the US population, independent of diabetes and hypertension [16]. One prospective cohort study on MetS in non-diabetic patients showed that MetS increased the risk for CKD [18]. Furthermore, a prospective cohort study on type 2 diabetes mellitus patients showed that MetS was associated with the CKD incidence, with central obesity, hypertriglyceridemia, and hypertension serving as independent predictive factors for CKD [16, 19].

A systematic review indicated that obesity was associated with CKD incidence and could increase the risk for CKD [8, 20, 21]. Additionally, a reduction in body weight could increase the eGFR in overweight males (BMI ≥ 25 kg/m^2^). Therefore, a reduction in body weight has kidney-protective effects in obese patients [22]. Studies by Kurella et al. and Chen et al. emphasized that obesity, especially the central obesity indicator waist circumference, was an independent risk factor for CKD [16, 23], which was consistent with our study results. The major pathology of obesity-related glomerulopathy (ORG) is glomerular hypertrophy, which can develop into secondary focal segmental glomerulosclerosis [24, 25]. The underlying mechanism is that adipose tissue synthesizes and secretes angiotensinogen, subsequently causing glomerular hypertrophy. Furthermore, insulin resistance increases type I and type IV collagen and fibronectin synthesis and induces segmental sclerosis, sclerosis of the glomeruli, and the development of obesity-related focal segmental glomerulosclerosis [26, 27].

Several limitations should be considered. First, the study population was selected from a rural area. Furthermore, the prevalence of cardiovascular events differs by region, ethnic group, and dietary habits in China [28, 29]. Therefore, the results from the present study cannot be generalized to the whole population. Second, the present study was cross-sectional; therefore, we cannot causally connect cardiovascular risk factors and eGFR. Third, although previous studies showed that abnormal blood glucose metabolism was associated with cardiovascular risk factors and a reduction in eGFR, our study did not show a correlation between an eGFR reduction and blood glucose, possibly due to the limited number of people with abnormal blood glucose in the selected population.

## Conclusions

Our study suggests that cardiovascular metabolic risk factors correlate with eGFR. Metabolic syndrome was associated with a reduction in kidney function and CKD.
